# Why Not the Best? How Science Failed the Florida Panther

**DOI:** 10.1371/journal.pbio.0030333

**Published:** 2005-08-23

**Authors:** Liza Gross

## Abstract

The US Fish and Wildlife Service is charged with using the "best available science" to manage endangered species. But a scientific review panel found "fatal flaws" in agency models used to regulate development in the habitat of the critically endangered Florida panther. What happened?

On a sultry spring night in 2001, wildlife photographer Brian Call was heading home from the back reaches of south Florida's Fakahatchee Strand. As usual, he saw no sign of the forested swampland's rarest inhabitants, the black bear and Florida panther—until he spotted a young panther, twisted and mangled in the middle of State Road 29. As Call sat beside the lifeless, still-warm body, he heard a chirping sound. “I finally realized,” he remembers, “it was the mother calling out.”

The mother's distress was even greater than Call imagined. The photographer laid the dead panther in the grass where he thought the mother was hiding, then reported the death to the state wildlife commission. Another panther had been run over just 300 feet away earlier that night, he was told. The panthers were ten-month-old siblings.

The Florida panther is one of the rarest mammals in the world, with an estimated population of 80 (Figure 1). About half of the panther's current range—just over 3 million acres—is on private land. The biggest threat to its survival is habitat loss. Southern Florida lost over 1.8 million acres of forest between 1935 and 1995 and gained 11,000 miles of public roads in just 12 years (1991 to 2003, the last year for which statistics are available). Vehicle collisions alone have killed 66 panthers since 1972, when the state started keeping track. Half of these deaths have occurred since 2000. But even more panthers die from wounds sustained during battles over ever-shrinking territories.

**Figure 1:**
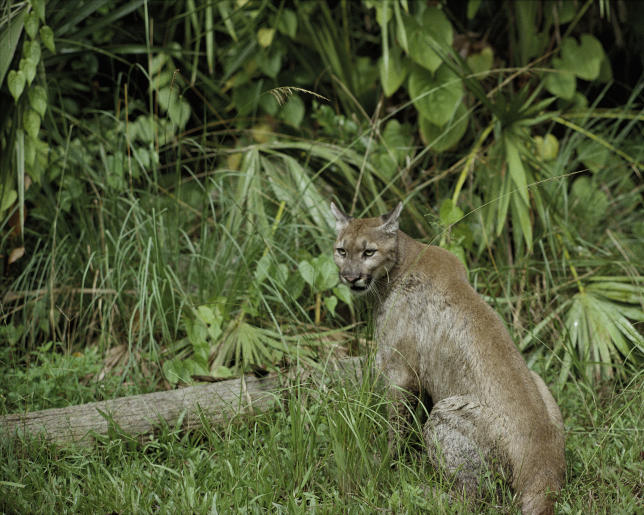
The Florida Panther National Wildlife Refuge Harbors One of the Rarest Animals in the World Just 80 Florida panthers remain in the wild—up from 30–50 animals in 1995. This critically endangered, wide-ranging carnivore faces the same problem that places endangered species at risk worldwide: fragmented, degraded, and vanishing habitat. Florida paves over 450 acres of green space a day, forcing panthers onto deadly roads and triggering often-fatal territorial battles over smaller and smaller ranges. (Photo: US Fish and Wildlife Service)

The fate of the panther rests with the US Fish and Wildlife Service (FWS), the federal agency charged with using the “best available science” to make sure the panther has enough habitat to find mates, hunt, disperse to new home ranges, and persist as a population. But the FWS was recently forced to acknowledge that, far from the best science, it has been using flawed science to regulate development in panther habitat. The agency's admission comes on the heels of rising tensions and internal disputes, which reached a crisis point soon after the formation of a new panther recovery subteam in 2000 (the panther subteam of Multi-Species/Ecosystem Recovery Implementation Team [MERIT]). Although most members of the MERIT subteam questioned the science behind FWS policy decisions, the agency had decades of peer-reviewed literature on its side, much of it published by a single researcher—David Maehr, a fellow recovery subteam member.

When the Scientific Review Team (SRT) analyzed 25 years and 3,000 pages of panther science, it discovered “fatal flaws” in FWS models used to evaluate habitat use and predict extinction risk. The FWS was making decisions that could place the critically endangered panther at risk without scientific justification.

The FWS continued to defend its policies despite the subteam members' vigorous protests and a lawsuit by environmental groups, which included court declarations from the dissenting team members. Finally, as arguments grew more personalized and entrenched—and after Andrew Eller, a veteran FWS biologist who worked on panther recovery for a decade, had publicly rebuked his agency for inflating panther numbers and misrepresenting the animal's habitat requirements—the agency called in an independent review team.

When the Scientific Review Team (SRT) analyzed 25 years and 3,000 pages of panther science, it discovered “fatal flaws” in FWS models used to evaluate habitat use and predict extinction risk. The FWS was making decisions that could place the critically endangered panther at risk without scientific justification. Unsound methodologies, the SRT reported, had passed peer review unchallenged. “Because of flaws in the system, we may have taken lands that we can't give back to the species,” says reviewer Howard Quigley, an expert on carnivore ecology and the executive director of Beringia South, an ecology research and educational institute.

Whether those “fatal flaws” had deadly consequences is in dispute. Meanwhile, scientists and policymakers must grapple with a more fundamental question: what happened to the “best available science”?

## Tracking a Secretive Species

In many ways, the story of the Florida panther (Puma concolor coryi) echoes that of every other large carnivore. Fearful settlers seeking revenge for lost livestock, and bounty hunters anxious to claim a $5 reward, killed panthers at every opportunity during the late 1800s and early 1900s. Extirpated from 95% of their historic range throughout the southeastern United States, panthers were reduced to a single, isolated population in southwest Florida by the 1950s (Figure 2). By 1967, state biologists thought they were gone there too. But in 1972, biologists found an old female, and the panther was listed as endangered when the federal Endangered Species Act (ESA) passed in 1973.

**Figure 2:**
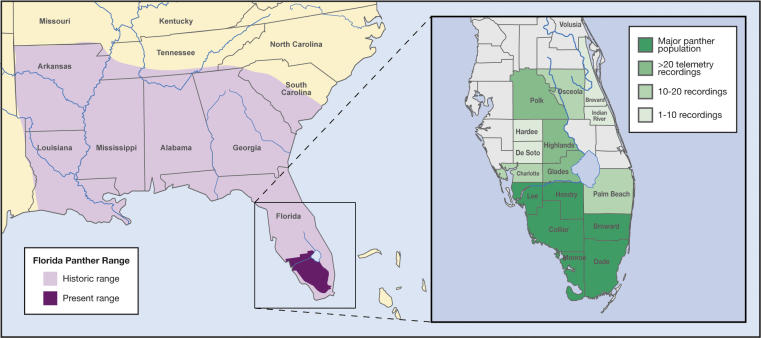
The Historic and Current Range of the Florida Panther The “Florida” panther is really a misnomer, experts say. This mountain lion subspecies once inhabited most of the southeastern United States, north to Tennessee and west to Texas—until human persecution and encroachment extirpated the panther from 95% of its historic range. The modern Florida panther is an artifact of these forces. Genetic drift and inbreeding led to the cat's trademark crooked tail and other unique characteristics, while unchecked development eliminated the panther from every area but southwest Florida—the last place to be developed. (Illustration: Rusty Howson)

When the Florida Fish and Wildlife Commission (FWC) formally started monitoring panthers in 1981, very little was known about the animal's biology. Because panthers, like cougars, pumas, and mountain lions (all describe the same subspecies), are shy and rarely seen during the day, capture teams used hounds to track their scent and chase them into trees. Once treed, cats were darted, collected with nets or rope, and then examined and tagged with radio collars while sedated. Radio telemetry provided data on location, range, behavior, and mortality (indicated by signal changes), and helped biologists study the health, social structure, and distribution of the population (Figure 3).

**Figure 3:**
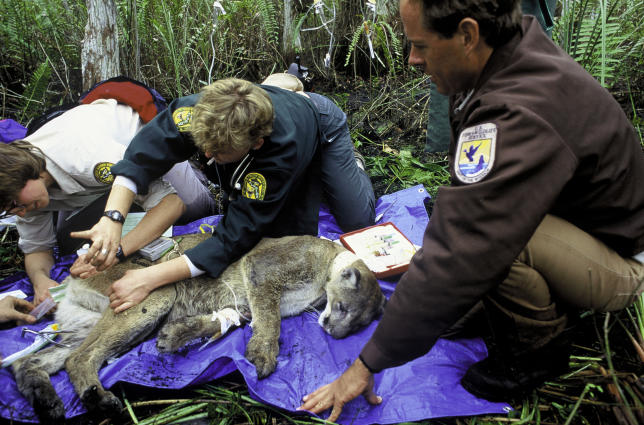
Collecting Valuable Data from a Sedated Panther After ensuring a panther has survived the ordeal of treeing and capture unscathed, a team of state and federal wildlife biologists examines the cat and collects samples for laboratory analysis. Cats are also dewormed and vaccinated. A saline drip keeps the animal hydrated while the team collects skin for genetic analysis, hair to measure mercury exposure, and blood to check for diseases. The FWS fears that a recent outbreak of feline leukemia in the Okaloacoochee Slough population could trigger accelerated extinction. (Photo: US Fish and Wildlife Service)

David Maehr inherited two radio-collared animals when he signed on as leader of the FWC project in 1985 and quickly established himself as the foremost panther authority. At that time, Maehr says, the population was considered old, parasite-infested, and ready to expire at any minute. When Maehr left the FWC in 1994 to work on his PhD, 54 panthers had been radio-collared, with about a dozen animals under study at a time.

Radio-telemetry data on 43 collared animals collected from 1981 to 1991 guided government agencies' first attempt to define panther habitat in a 1993 habitat preservation plan. To qualify as high-quality habitat, an area had to be large enough to “support several panthers.” It also had to be contiguous with occupied range and contain significant forest cover, few residences, and few highways. Based on the telemetry data, forests got highest priority while less-forested public lands in Big Cypress National Preserve and Everglades National Park were considered less suitable. The notion of forest dependency was further developed by Maehr and James Cox, in a 1995 peer-reviewed *Conservation Biology* paper that would lay the foundation for future FWS decisions in panther habitat.

This paper and the inferences it supported placed Maehr at the center of controversy and eventually cost Andrew Eller his job. And when the SRT reported that the “most influential paper on panther habitat use” contained four fatally flawed inferences, the scientific foundation on which an imperiled species's survival depended began to crumble.

“It was put in very strong language that panthers are forest obligates and wouldn't move across more than a 90-meter (about 300 feet) gap of non-forest,” marvels SRT reviewer Paul Beier, a mountain lion expert. Considering that the average home range for a male is 200 square miles, Beier adds, “this was a very improbable conclusion.”

Maehr and Cox's conclusions were problematic for a variety of reasons. The researchers presented 14,500 locations from 41 radio-collared panthers, but analyzed only 8,600 locations for 23 animals—without acknowledging doing so. “They excluded animals that were out in the swampland and then came to the conclusion that panthers only used forest,” Beier says.

It was put in very strong language that panthers are forest obligates and wouldn't move across more than a 90-meter (about 300 feet) gap of non-forest....this was a very improbable conclusion.”

Also at issue was the telemetry data itself. “They were taking telemetry observations from airplanes with known positional errors on the order of 200 to 300 meters or more and then making claims about habitat use at 100 meters,” says reviewer Michael Conroy, a population ecologist with the US Geological Service in Georgia. “You simply can't do that.”

But the biggest problem, says reviewer Michael Vaughan, was that all the readings were taken during the day, and panthers are nocturnal. “Most of their feeding activity is at night, they're hunting at night, and using other areas that they don't use during the day,” says Vaughan, an expert on large carnivore ecology with the US Geological Service in Virginia. Maehr and Cox extrapolated their daytime data to 24 hours a day, he explains. And, echoing Conroy, he adds: “You can't do that.”

“It's unfortunate that this paper was published in a peer-reviewed journal,” Vaughan says. “Once it's peer-reviewed people tend to take it as gospel. But the peer-review process was bad.”

Since Maehr either authored or coauthored some 75% of the habitat-related research on the Florida panther, his reputation has taken a beating and his work has lost credibility. He admits that mistakes were made in data analysis, but defends his conclusions. “One of the studies that we did in terms of great cost in manpower and discomfort in the field was to go out and actively monitor animals around a 24-hour period,” he says. By counting the tip-switch changes in radio collars, Maehr says, he could tell whether an animal was active or not, and found that panthers are crepuscular—most active around sunrise and sunset.

Maehr says there was nothing to suggest that panthers abandoned places they occupied during the day or exhibited patterns different from those suggested by “tens of thousands” of radio locations collected during the day, which he says included the panther's most active periods. “The bottom line is that those data do reflect a 24-hour cycle of activity and habitat use.”

One of the researchers on the MERIT panther subteam, however, argues that the 24-hour monitoring study showed only peaks of activity and did not identify habitats associated with locations. “Tip-switch data record head movement,” says Jane Comiskey, a researcher at the Institute for Environmental Modeling at the University of Tennessee and a vocal critic of Maehr's work. In confined trials, she explains, many times walking isn't recorded because there is no head movement; on the other hand, many activities that accompany head movement, such as feeding and grooming, occur when the animal isn't walking.

Despite all the problems in the 1995 paper, its conclusions—panthers are forest obligates that will not cross 90 meters of unforested landscape—would form the cornerstone of a habitat evaluation model used during FWS development permit consultations. Since 1995, the FWS has approved permits for 35 development projects impacting 38,484 acres in and around panther habitat.

## Deconstructing the Panther Landscape

Under Section 7 of the ESA, federal agencies must consult with the FWS to make sure their activities don't jeopardize the habitat or survival of threatened or endangered species. In panther country, most consultations involve US Army Corps permits to destroy wetlands for urban and agricultural development. The FWS writes biological opinions that assess the impacts of development and identify ways developers can mitigate these impacts—by preserving acreage elsewhere on the property or offsite, for example. In 1998, Eller and his coworkers had determined that a Lee County, Florida, Department of Transportation project called the Daniels Parkway extension would degrade 1,540 habitat acres and that the county could mitigate by preserving 250 acres elsewhere. The county disagreed, and in 1999 paid $317,000 to Dawson and Associates, a Washington, D. C., lobbying firm that represents Florida development and agricultural interests, to “advise and assist Lee County in obtaining more timely resolution and significantly more reasonable terms in the federal permit for [the] Daniels Parkway extension,” according to a purchase order obtained from Lee County.

That same year, during a Section 7 consultation on Daniels Parkway, Maehr presented a new habitat evaluation model to the FWS. Maehr, working as a consultant for Lee County, developed the model with Jonathan Deason, a professor of environmental and energy management at George Washington University. Deason is also listed as a senior advisor to Dawson and Associates. The model, called the Panther Habitat Evaluation Model (PHEM), estimates functionally equivalent panther habitat units based on weighted scores for six habitat factors. The PHEM score determines habitat impacts and corresponding mitigation. Seventy-five percent of the score is based on four factors: land vegetation type (hardwood hammock forest gets the highest value), forest patch size, proximity to forest (the 90-meter rule), and proximity to a population core. All four factors were based on what the SRT called flawed inferences in the 1995 paper.

The same week PHEM was presented, Senator Slade Gorton (Republican, Washington)—a proponent of scaling back ESA habitat protections—quietly inserted a rider into the 2000 Department of the Interior appropriations bill directing the FWS “to work cooperatively with the Lee County, Florida, Department of Transportation to ensure that measures designed to minimize the impacts on the Florida panther related to the Daniels Parkway extension are reasonable and conceived properly.”

Although the language in the rider “sounded innocuous,” Eller says, “we knew it meant accept PHEM or else.” When FWS biologists were asked to evaluate PHEM, Eller says, they decided it should not be used in permit decisions. A major problem with PHEM is that it starts with an ideal 500-hectare (two square miles) patch of oak hammock that's less than 90 meters from the nearest forest patch, says Eller. “If habitat didn't match that ideal, then the score was lowered. But nothing in the areas of concern matches that description.”

The FWS biologists wrote a letter intended for Maehr explaining the problems, Eller says, but the letter wasn't sent until about three months after consultation on Daniels Parkway concluded. In the end, based on PHEM, the agency preserved just 94 acres—less than 40% of what the biologists recommended based on their own models.

Essential steps were skipped by developing an impact-assessment model based on daytime panther locations, observes Comiskey. Because only the most frequently used lands merit mitigation under PHEM, much of the forest of southwest Florida didn't qualify. “In the Daniels Parkway extension, only 11% of the forest on the project site qualified based on the rules for patch sizes, proximity to other patches and the [population] core, forest type, and connectivity,” Comiskey says. And because of the 90-meter rule, no mitigation was required for the largest forest patch.

“It's damaging to the credibility of the authors, the journals, and the reviewers who approved the papers to have published sensitive methodologies that could put a highly endangered animal at risk without scientific justification,” Comiskey says. “Especially when potential conflicts of interest are involved.”

The unreliable inferences about panther demography “muddled the debate” about the viability of the population, the SRT wrote, adding “unwarranted credibility” to arguments against genetic restoration.

Maehr brushes aside suggestions of conflict, insisting that he worked with developers to find creative ways of dealing with development and to convince landowners that having a panther on their land would be an asset, not a liability. “You're not going to make it an asset by pounding private landowners over the head with the panther.”

And Maehr says he never intended that PHEM become the “agency standard” for permit review: “We viewed it as a step in a process that would lead to a better approach.” He concedes that the 1995 paper failed to explain why Everglades panthers were excluded. But it was reasonable to do that, he argues, because the Everglades population appeared to be in the “final throes of extinction” and the open expanses of southeastern Florida were “atypical and unsuitable” habitat.

Analytical problems aside, Maehr defends his conclusions. “All evidence continues to point to the critical nature of forest,” he says, adding that it's dangerous to say that areas with little forest, like the Everglades, provide “excellent” panther habitat. “This almost jihad-like insistence that panthers are generalists is [argued] so the panther becomes this wonderful umbrella to block any efforts to continue to develop south Florida,” Maehr says.

Yet arguing that the panther is a forest obligate, critics say, safeguards only forest. “We all agree that removal of forest impacts panthers,” Comiskey says. “What we disagree about is whether development of other land covers also impacts panthers.”

In southern Florida, forest cover often occurs in scattered tree islands separated by open prairies, marshes, shrubland, and saw palmetto thickets interspersed with agricultural and improved pasture lands. “I went into consultation after consultation arguing with both consultants and the [US Army] Corps who said that the only habitat that needed to be compensated was forested,” says Eller.

In 2002, Eller began to accuse his agency of using flawed science and pressuring biologists to rewrite biological opinions to favor development. Development is outpacing habitat protection by one and a half times, Eller says. When he noted that trend in a biological opinion, however, “they took that information out.”

With help from Public Employees for Environmental Responsibility (better known as PEER), Eller sued the Department of the Interior (the FWS is a bureau in this department) under the Data Quality Act in June 2004 for using unsound science. That November, after 17 years with the FWS, Eller was fired for not meeting deadlines and “unprofessional exchanges with the public,” among other charges.

Since 1999, the FWS has issued 29 biological opinions for development permits in or near panther habitat. “A lot of land was developed that likely would not have been developed had these flawed inferences not appeared in those publications,” says SRT reviewer Vaughan. “And they were calling this the ‘best available science.’”

In March, outgoing FWS Director Steve Williams upheld Eller's data quality challenge, and admitted the agency used flawed science. In a letter to PEER, Williams wrote that he ordered the FWS regional director to “immediately update the panther-related sections” of a multi-species recovery plan and “incorporate appropriate recommendations of the SRT.” Williams also acknowledged that the agency mistakenly equated verified population with minimum viable population—the number required for persistence. Again, these assumptions were traced to one journal article. In June 2005, Eller was reinstated by the FWS, after both parties reached an out-of-court agreement.

## Driving the Panther into a Genetic Bottleneck

Until the early 1900s, the Florida panther (P. concolor coryi) crossbred with the Texas puma (P. concolor stanleyana), along with two other now-extinct races, where their ranges once overlapped. (The Florida and Texas subspecies shared a common border along the western edges of Louisiana and Arkansas.) Once isolated, panthers suffered inbreeding depression. Some external anomalies like cowlicks and crooked tails aren't likely to have serious effects on fitness. But heart defects, susceptibility to heavy parasite loads, and reproductive abnormalities—including poor sperm quality and undescended testicles—could prove devastating. “Geneticists were telling us the cats were so inbred they would probably only survive another 25 to 40 years,” says Eller. Population estimates in the 1990s hovered around 30 to 50 animals.

In an effort to revitalize the population, the FWS released eight female Texas cougars into unoccupied panther ranges in 1995. But a group of papers on panther genetics and demography questioned the need for introducing outside genes, called genetic introgression. The papers claimed that even though observed defects likely had a genetic basis, they had minor impacts on individual and population fitness. The introgression experiment generated significant controversy, SRT reviewer Quigley says, with one side saying the population was vigorous and a variety of others saying it wasn't and that introgression was critical to the panther's survival. There's a legitimate argument to be made against introgression from an evolutionary standpoint, Quigley says: “If there's any indication that an endangered species can be recovered without watering down the original genetic stock, then we should be willing to do that.”

But in this case, he says, the question boiled down to whether the population was on the verge of extinction and needed introgression as a last resort. And the goal of the introgression experiment was to mimic historic gene flow, not to replace or swamp the panther gene pool. The argument against introgression suggested the population was vigorous and so introgression was unwarranted. “The claims were that the populations were doing pretty well prior to the introduction of Texas cougars,” says the SRT's Conroy.

The review team traced evidence for arguments against genetic introgression to “one graph and four sentences” in a 1995 *Conservation Biology* paper coauthored by Maehr and Gerard Caddick. In the paper, the authors interpreted a figure that plotted births against deaths per year as evidence of a robust population. But the SRT found a problem with mortality detection (kittens under four months weren't radio-collared and couldn't contribute to total deaths). And because it wasn't clear that births and deaths (presented as raw numbers) corresponded to the same population base, the SRT concluded, the graph couldn't support inferences about per-capita vital rates or population growth rates.

Growth-rate estimates are one parameter used in population viability analyses (PVAs) to predict extinction risk. Another is kitten survival—estimated at 84% to 87% in the Maehr and Caddick paper. The SRT called the estimate—presented with no underlying data—“indefensible,” citing a more recent data-supported estimate of 52% for pure panthers. The team also wrote that it would be unprecedented in vertebrate studies for kitten survival to exceed adult survival, which was estimated at 82%. (Maehr sees no problem with kitten survival exceeding adult survival. “We were talking about kitten survival through one year of age, when they're protected by their mothers.”)

The kitten survival estimates were used in a PVA—peer reviewed and published in a 2002 University of Chicago book—that the FWS used to assess panther viability. Using the unsupported kitten survival rate, the 2002 PVA estimated extinction risk at 0%. The analysis also failed to model the effects of changes in habitat or genetic restoration on extinction risk, the SRT reported, assuming they would have none. “Clearly,” the team wrote, “some combination of these changes caused extinction risk to plummet from 100% to about 0%.” It was this model that Eller cited when criticizing his agency for “pretending there's a surplus of panthers.”

The unreliable inferences about panther demography “muddled the debate” about the viability of the population, the SRT wrote, adding “unwarranted credibility” to arguments against genetic restoration. By most accounts, the cross-breeding experiment reinvigorated the population. Hybrids have a higher kitten survival rate than pure panthers, and hybrids show a dramatic decrease in the prevalence of abnormalities. “So far it looks like this experiment is working,” Quigley says, “but the review team felt there wasn't enough genetic monitoring going on.”

The SRT report urges management agencies to collect genetic data and study the impacts of genetic restoration on individual traits and demographic vigor. “The Florida panther story promises to be the best documented example of the relevance (or irrelevance) of genetics to conservation,” the team wrote. “Future conservation decisions deserve to be informed by the results of the panther introgression experiment.”

## Recovering the Panther—And Scientific Integrity

In June, a healthy three-year-old male panther was killed on an interstate highway in northeastern Florida, 350 miles from known panther range. FWC officials think he wandered up from south Florida in search of a mate. “With more roads, more people, and more development, panthers are being squeezed out of their ranges and forced to go north,” says FWC spokesperson Karen Parker.

Panther habitat is shrinking at a rate of nearly 1% a year. If that trend continues, 500,000 more acres—over 15% of all remaining panther habitat—will vanish in 25 years. Nothing suggests the trend will change (Figure 4). In 2006, developers hope to finish construction on a new city and Catholic university called Ave Maria. The project, which the FWS estimates will impact nearly 5,000 acres, is sited right next to the Florida Panther National Wildlife Refuge along SR29, where photographer Brian Call found the road-killed panther. “If the FWS isn't going to write a jeopardy opinion or recommend the [US Army] Corps deny a permit for something like Ave Maria,” says Eller, “the sky's the limit.”

**Figure 4:**
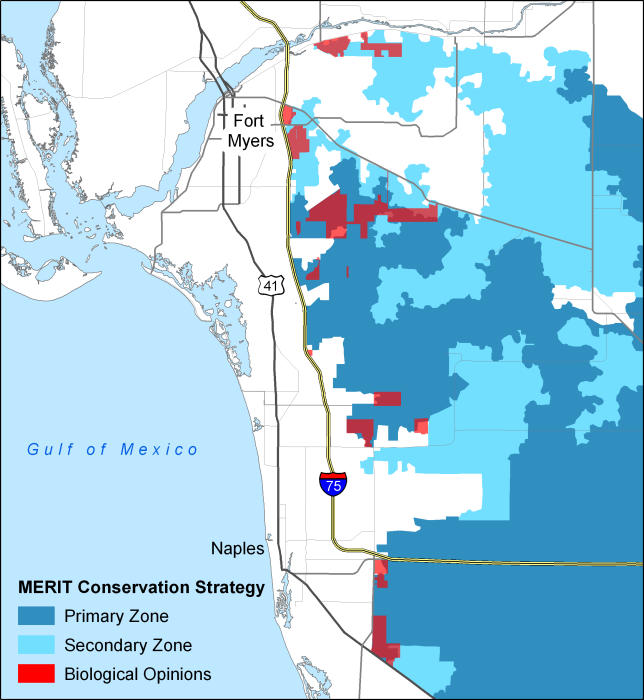
Development Projects in Panther Habitat Since 2000 In a 2002 landscape conservation strategy, members of the MERIT panther recovery subteam identified lands essential to the long-term survival of the Florida panther. The MERIT subteam defined the Primary Zone (dark blue) as “all lands essential for the survival of the Florida panther in the wild.” The Secondary Zone (light blue) includes “lands contiguous with the Primary Zone, and areas which panthers may currently use, and where expansion of the Florida panther population is most likely to occur.” Maintaining the integrity and connectivity of the Primary Zone, which includes both public and private lands, is one of the highest priorities for panther survival, the subteam determined. It also stressed the importance of avoiding “land-use intensification” in the Secondary Zone, as well as in areas that panthers use to disperse. Yet development continues. Since 2000, the FWS has written 27 biological opinions (red) evaluating the impacts of Florida development projects in panther habitat. (This map does not include biological opinions written in 2005.) (Illustration: Brad Nunley/National Wildlife Federation)

When a population drops below 500, conservation biologists start to worry about extinction. With fewer than 100 panthers left, every animal counts. Yet the FWS assumed there were plenty of panthers and development could proceed anywhere except forests. How could the scientific review process go so wrong with so much at stake? “We just were not policing ourselves well,” says the SRT's Quigley. “Up until seven years ago there wasn't anyone questioning the science that was coming out in the publications. There was just one very prolific scientist.” What scientists should be doing is developing new knowledge and adjusting current views in a healthy exchange of ideas, he adds. “What we see in this case is a program that became so entrenched in territoriality that the kind of vibrant free exchange of ideas just didn't happen.”

California law requires independent scientific review at several stages of the conservation planning process, “so consultants working for counties or developers are not able to get away with using flawed scientific methodologies,”

While peer review is not without its problems—unsound interpretations and conclusions slip through the process from time to time—the case of the panther is unusual in that the peer-review process failed to catch such seriously flawed inferences in science used to manage an endangered species. When the two *Conservation Biology* papers were published in 1995, though Maehr was considered the panther expert, he had only just started his doctoral work. Why didn't more senior scientists flag the problems in the review process? “Peer reviewers have to rely on what the authors report in their paper,” says Reed Noss, editor of *Conservation Biology* at the time the papers were published and Davis-Shine Professor of Conservation Biology at the University of Central Florida. “Ultimately the onus is on the authors to use all the available data [for example] or explain why certain data were excluded from the analysis.”

“I think there were definitely questionable interpretations of the data,” Noss says. “Whether it was intentional or just a mistake, it's hard to say.” But peer review will never be able to uncover all these kinds of methodological problems, Noss explains, because with so much competition for space in the journals the details of the methods don't appear in the articles. “The word limits keep getting lower and lower. You would have to do what this independent team of reviewers did and go to the original data. There's no way a peer reviewer is ever going to have time to do that.”

The shortcomings in peer review underscore the importance of having open review and independent scientific review panels like the SRT, Noss says, and like those mandated by California law. Unlike the federal ESA, California law requires independent scientific review at several stages of the conservation planning process, “so consultants working for counties or developers are not able to get away with using flawed scientific methodologies,” says Noss. “And quality control is assured all the way through the process.” It's what the agencies should have been doing all along for the panther, he adds. “They shouldn't have waited until a couple of years ago to have an independent scientific review of such a high-profile, controversial, and highly endangered species.”

So why doesn't the FWS vet the science it uses to manage endangered species as a matter of course? “We certainly have a vetting process, but science evolves, so we have to constantly reevaluate what we use,” says Paul Souza, an FWS assistant field supervisor. “We can always do better, but our process is a good one. Our biologists are on the front lines of research and with the help of these groups like the SRT and MERIT, we're identifying problems and gaps in the science,” Souza says. “We just revised a biological opinion on Ave Maria when we realized we had [underestimated] the value of a panther corridor important for dispersal along Camp Keais Strand,” he adds. The project will change a two-lane rural road into a four-lane urban highway. In the revised opinion, the FWS determined that a wildlife crossing should be built to offset the expected impacts of increased motor traffic and reduce the number of road-kill incidents. Souza says the agency will soon begin meeting with FWC officials and panther scientists to review the SRT recommendations and determine what findings should be incorporated into agency decisions.

To guard against future breakdowns in the system, the SRT wrote an article recommending ways to improve peer review and ensure science-based conservation. The team also wrote a summary of the problems found in the panther literature. Both reports are in press at the *Journal of Wildlife Management*. One recommendation involves using an independent scientific advisory committee to vet the credibility of each piece of evidence used in a recovery effort, says Quigley. “That was not done with the Florida panther, and you can see where it ended.”

Many flaws can be corrected by rigorous reanalysis of existing datasets. “There's a difference between being wrong and being unreliable,” SRT reviewer Beier says. “The scientific process that yielded certain conclusions wasn't sound. We're not sure whether the conclusions are right or wrong.” A robust model for evaluating panther habitat use can be developed from reanalyzing the existing data, he says, including the data omitted from panthers in the Everglades, and by collecting better data on nighttime use. And better measures of reproductive success and age-specific survival will yield better population models, says reviewer Vaughan. “We need to go back and get more data to get a better handle on kitten survival. Then you can model population growth and reproductive rates.”

One thing is certain. The panther will not survive if it is relegated to a single, isolated population in south Florida. One of the most urgent needs is to identify some of the best release sites for panther reintroduction, Beier says, “develop a plan for release, and follow through on it. We need some science for that and we need a whole lot of political will.”

Developing reliable methods of studying low-density, elusive carnivores like the panther will help wildlife biologists better understand and manage other endangered species. And preserving habitat for the Florida panther provides protection for all the species that share its range—a group that includes 52 state and federally listed species. “Habitat conservation does require that certain areas don't turn into housing tracts,” says Beier. That's why it's essential to start with a sound scientific foundation. Once you build a shopping mall or golf course in prime habitat for a wide-ranging species, you can't reclaim that land if you discover you made a mistake. Everyone knows the panther needs habitat more than anything else, Quigley says, so “it's best to err on the conservative side” when mistakes could threaten the survival of a species. “And that's why it's so vital to fix the flaws in the system,” he says. “So it won't happen again.”

## Abbreviations

ESA: Endangered Species Act
FWC: Florida Fish and Wildlife Commission
FWS: US Fish and Wildlife Service
MERIT: Multi-Species/Ecosystem Recovery Implementation Team
PHEM: Panther Habitat Evaluation Model
PVA: population viability analysis
SRT: Scientific Review Team
